# Spontaneous perceptual reversals are accompanied by systematic changes in pupil size but not respiration phase

**DOI:** 10.1038/s41598-026-53388-5

**Published:** 2026-06-05

**Authors:** Lisa Stetza, Christoph Kayser

**Affiliations:** Department for Cognitive Neuroscience, Faculty of Biology, Bielefeld University, Bielefeld, Germany

**Keywords:** Respiration, Respiration phase alignment, Bistable object, Perceptual reversal, Pupil size, Neuroscience, Physiology, Psychology, Psychology

## Abstract

Humans tend to align their respiration with important events, in line with the notion that respiration serves as a tool to allocate (neuro-)physiological resources. This respiration alignment is consistently reported in laboratory studies relying on sequences of temporally structured, stimulus-driven experimental trials. Whether this alignment also occurs in the absence of external cues - such as during spontaneous changes of perception - remains unknown. Notably, pupil size is known to change around spontaneous changes in perception, and pupil size covaries with respiration phase, suggesting that respiration may also align to spontaneous changes in perception. We here investigate the three-fold relations between respiration, pupil size, and spontaneous perceptual changes in a paradigm where participants (*N* = 20) reported spontaneous perceptual reversals of an ambiguous Necker cube. We confirmed the biphasic modulation of pupil size around reversals (constriction followed by dilation) and the covariation of pupil size with respiration phase. However, the alignment of respiration phase to perceptual reversals was much weaker than previously reported for stimulus-driven experimental trials. These findings suggest that changes in perception alone, whether exogenously or endogenously driven, might not be the key driving force of respiration alignment, but the temporal predictability of external events likely facilitates this alignment.

## Introduction

We tend to focus our respiration on salient events. In typical sensory-cognitive lab studies, for example, participants tend to align their respiration with the expected timing of individual trials and this alignment is predictive of how accurate or fast participants respond^1–5^. At the same time, neural activity covaries with respiration. This includes a widespread modulation of brain activity with respiration at rest^6–8^ but also entails the covariation of neural activity directly relevant to the current task^1,5,9–12^ and of activity reflecting arousal or attention with the respiration cycle^10,11,13–15^. This suggests that by aligning respiration with temporally expected sensory-motor events, the brain can ensure the optimal handling of these^6,16,17^. Despite many studies supporting the alignment of respiration to external events^1,2,4,5,9,11,18–24^, it remains unclear whether an alignment of respiration to perceptual-motor events also extends to situations where external cues are absent, such as during spontaneous changes of (bistable) perception.

Yet, such spontaneous changes in perception provide an important testbed to understand what factors drive the alignment of respiration. Previous studies on respiration alignment confound different putative drivers of this: one driver in these studies could be the expectation of specific externally timed requirements to act on sensory-motor contingencies, such as the expectation of an upcoming stimulus or the need to act on this^2,10,24,25^. In this case the respiratory alignment would be tied to temporally expected and external events and may be absent during purely intrinsically triggered actions. An alternative driver in these experiments could be the intrinsic coupling of task-relevant neural processes to respiration, such as processes reflecting the encoding of newly received sensory signals. In this case, the engagement of these processes at a specific moment of the experimental trial results in an alignment of respiration with the external event. Only in the second case would one expect an alignment of respiration to spontaneous changes in perception, while the need for externally controlled expectations or sensory-motor contingencies would not predict an alignment to spontaneous changes in perception, as discussed in the following.

Indeed, the coupling of neural activity to respiration extends from sensory to association regions and likely includes those regions involved in mediating spontaneous changes in perception, such as temporal, parietal and prefrontal cortex^26–29^. Any modulation of neural processes in these regions along the respiration cycle could result in the spontaneous changes in perception to covary with the respiration phase, for example, by adjusting the gain of individual sensory representations or biasing decision processes along the respiratory cycle towards one interpretation of a bistable stimulus. Further support for a potential alignment of respiration to spontaneous changes in perception comes from studies showing that pupil size changes around spontaneous perceptual reversals^30–33^. Pupil size is a key marker of arousal^34–36^ and has been shown to systematically decrease prior to and increase after perceptual reversals^32,33,37^. This alignment of pupil size to perceptual reversals may reflect transient changes in neural representation of the ambiguous object (constriction) as well as motor activity when reporting the reversal (dilation)^31,32^. At the same time, pupil size was shown to change systematically along the respiration cycle^13,38^. Together this may reflect a common covariation of respiration with changes in arousal and spontaneous changes in perception.

Alternatively, respiration may align only to externally predicted events, be it the expectation of a particular sensory stimulus or the expectation to perform a specific motor behavior^21,39^. In the absence of such events, the respiration alignment would vanish. The absence of alignment of respiration to spontaneous changes in perception could also be motivated by the observation that the time scale of neural activity underlying the encoding of typical external stimuli differs from that observed during intrinsic changes in perception. The neural responses evoked by external stimuli are typically characterized by a fast rise and a slow decay (peak latency between 50 and 200 ms after stimulus onset for visual or auditory stimuli) and usually emerge from early sensory areas whose neural activity varies on a fast time scale^40,41^. In contrast, the neural activity associated with endogenous perceptual changes seems to emerge across multiple and mostly slower time scales, as suggested by studies on binocular rivalry and visual bistable perception. These reported slow and ramping changes in activity over 1–2 s before the endogenous perceptual change, arising gradually from a hierarchy of brain regions^40,42,43^. Following this line of thought, one may not necessarily expect a relation between respiration and spontaneous changes in perception.

To address this question, we tested whether the respiration of human participants aligns to spontaneous changes in the perception of a bistable visual object. In our study, participants viewed the Necker cube, an ambiguous figure depicting a cube that can be interpreted as being seen either from the left or right side. This figure has two perceptual interpretations (bistability), although it does not change physically. We presented this figure for prolonged periods (75-s trials) and participants reported their perceived perceptual reversals using a button press, while we recorded respiration and pupil size. We then tested whether respiration specifically aligns to perceptual reversals, whether reversals emerge during a particular respiration phase, and whether the duration of individual periods of perceptual stability is related to respiration phase or rate. In addition, we confirmed previous results showing that pupil size systematically changes around perceptual reversals and confirmed that pupil size systematically covaries with the respiration phase^13,38^.

## Methods

### Participants

A total of 32 adult volunteers participated in the study after providing written informed consent. All participants self-reported normal or corrected-to-normal vision and hearing and were compensated for their time; participants consisted of typical young university students (demographic data were not recorded). Participants were informed about the experimental procedures and devices but were not explicitly told that the study focused on the relationship between respiration and task performance, similar to our previous work^2,4,5^. They were instructed to breathe normally through their nose, though we cannot exclude that some participants also breathed orally during the task. The Ethics Committee of Bielefeld University approved the study. Data acquisition was performed in accordance with the Declaration of Helsinki.

### Experimental setup and data acquisition

The experiment was conducted in an electrically shielded and sound-attenuated booth (Desone, Germany). Visual stimuli were presented on a computer monitor (27 inch; ASUS PG279Q, 120 Hz) positioned approximately 85 cm from the participant. Visual presentation was controlled using MATLAB (R2017a, MathWorks Inc., Natick, MA) and the Psychtoolbox (Version 3.0.14). During the experiments there was no additional light than the monitor. Synchronization of stimulus presentation and respiration recordings was implemented via TTL pulses sent to the BioSemi ActiveTwo system (ActiView software).

Respiration was recorded using a temperature-sensitive thermistor (Littelfuse GT102B1K, Mouser Electronics) attached to a modified single-use oxygen mask^2,4,5^. The changes in voltage generated by temperature fluctuations from nasal airflow during inhalation and exhalation were amplified and recorded via the analogue input of the BioSemi EEG system. Eye movements and pupil diameter were recorded monocularly (left eye), while participants viewed the cube binocularly, using an EyeLink 1000 (SR Research Ltd., Canada) at 500 Hz in a free-viewing configuration. A 9-point calibration was performed prior to each block. Pupil size was measured using the systems Ellipse algorithm.

### Experimental task

**Fig. 1:**
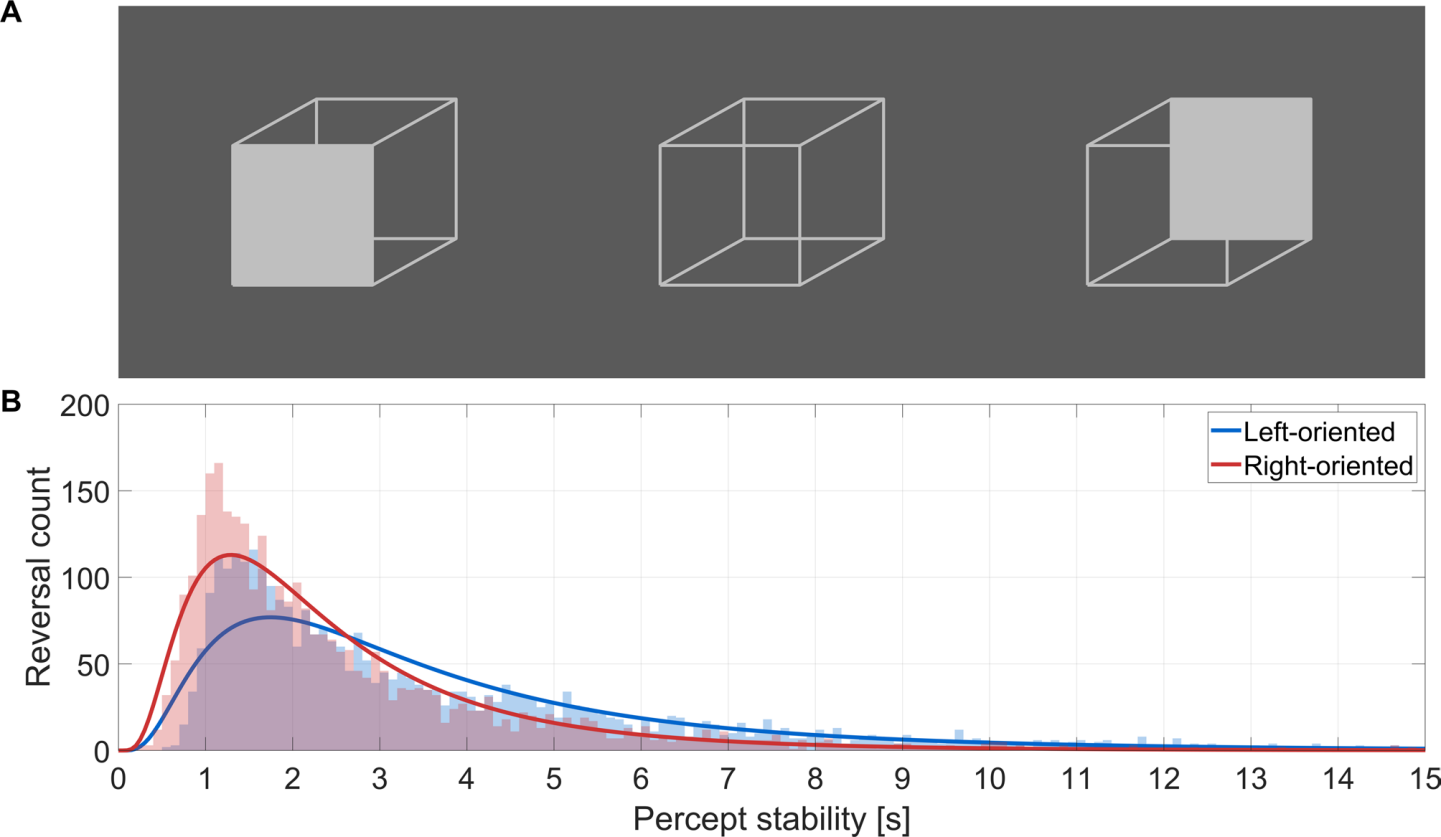
Testing perceptual stability. (**A**) A disambiguated version of the Necker cube was shown to participants during the familiarization phase to associate each interpretation with the respective response key (left and right panels). During the experiment participants saw an ambiguous version (middle panel) for continuous periods of 75 s, while reporting spontaneous reversals in their perceived interpretation of the cube. (**B**) Distribution of percept stability for the left-oriented and right-oriented interpretation of the cube, across participants (*N* = 20). Curves show the best fitting lognormal fit.

The Necker cube covered 11°x9° of visual angle (line width 3 pixels, color RGB 115 115 115) and was of comparable size to previous studies^27,44,45^. The background of the screen was uniform grey (RGB 70 70 70; 18.5 cd/m^2^) and constant throughout stimulus and inter-trial periods to avoid changes in luminance. Participants were first familiarized with the experimental task to ensure they understood and experienced spontaneous reversals of the Necker cube. For this, disambiguated variants of the cube with one face shaded in grey were presented to associate these with the respective response buttons (left and right arrow keys) (Fig. 1A). During the actual task, participants completed 24 trials, each consisting of 75 s of continuous stimulus presentation, with their task being to report whenever the percept changed by pressing the corresponding button. Each trial was followed by a 15-s inter-trial interval. The 24 trials were grouped equally into four blocks separated by breaks. During each block, the luminance of the screen was constant and participants were adapted to the darkness prior to starting each block to avoid any changes in pupil size associated with changes in ambient luminance.

Participants were instructed to keep their gaze on the screen to prevent missing trial on- and offset, but were otherwise allowed to gaze freely, also to prevent fatigue from continuous fixation. Participants were kept as naive as possible to the task and were not instructed to look at particular areas of the cube or to intentionally provoke reversals.

### Processing of respiratory data

Similar to our prior work^2,4,5^, the respiration signal was preprocessed using FieldTrip^46^ in MATLAB (R2022b). Signals were filtered using a third-order Butterworth filter (high-pass at 0.03 Hz, low-pass at 6 Hz), resampled at 100 Hz and converted to z-scores. The Hilbert transform was used to compute the analytic signal, from which local peaks were identified to define individual respiration cycles. Cycles were extracted in 7-s windows centered on these peaks, including only those peaks exceeding z = 0.5. Inspiration was defined based on a positive slope prior to a peak, and expiration based on a negative slope following this. In some cases, short pauses between inhalation and exhalation were classified as atypical data points and not assigned a specific phase^47^. We also identified atypical respiration cycles by calculating the mean squared distance of all respiration cycles of each participant in time; cycles exceeding 3 standard deviations from the centroid were removed from data analysis. To link respiration to behavior the respiration phase was defined as a linear variable progressing from 0 to π during inspiration and π to 2π during expiration. This approach provides a continuous representation of the respiration phase with meaningful reference points (0/2π = peak inhalation; π = peak exhalation). We also determined the respiration frequency prior to each perceptual reversal. We defined this based on the average of the instantaneous frequency of the last two cycles prior to each button press. As a control, we also defined the respiratory frequency based only on the last cycle prior to each button press. We then calculated the within-participant correlation of these two definitions of respiration frequency, which was high 0.78 ± 0.07 (SD), suggesting that using either definition should result in similar conclusions.

### Processing of eye tracking data

The pupil diameter was extracted using custom MATLAB scripts and obtained following previous work^32^: periods of missing data (blinks) were identified and linearly interpolated. Any potential temporal drift of pupil data was corrected by fitting an exponential function to the data and subtracting this fit from the original signal. To reduce noise and artefacts, the detrended signal was low-pass filtered at 8 Hz. For alignment with respiration data, the filtered and smoothed signal was resampled to 100 Hz.

### Data exclusion and cleaning

The inspection of the behavioral data revealed a large inter-individual variability in the number of perceptual reversals, ranging from fewer than 60 to more than 1200 reported reversals. Such variability can complicate data analysis and the comparison with previous studies. To standardize the dataset, we excluded participants who reported more than 900 reversals (i.e., one every 2 s) or reported fewer than 120 reversals. The former criterion was implemented as a very high number of reported reversals is somewhat incredible based on previous studies^27,33,43^, and does not allow discounting effects of previous or subsequent reports in the data epochs cut around each reported reversal (see below). The latter criterion removes those participants that effectively had offered too little data for analysis. Based on these criteria, we excluded data from 10 participants (3 exceeded the maximum, 7 fell below the minimum). For some participants the eye tracking did not work reliably during some of the experimental blocks leading to the loss of some data. For the final analysis we stratified the dataset to include only those participants with robust behavioral data, respiratory data and eye tracking data. Specifically, for the analyses linking respiration phase and behavior, we retained data from *N* = 20 participants, from which we included on average 334.9 ± 178.8 (mean ± SD) reversals (reflecting 81.8 ± 12.6% of the reported reversals). For the analyses linking behavior, respiration and pupil size, we included only reversals with reliable eye tracking data resulting in 319.6 ± 170.6 reversals per participant (reflecting 79.5 ± 10.2% of the reported reversals).

To test whether some of the reported results are influenced by the occurrence of another indicated reversal in the 4 s data epochs cut around each reversal, we intended to repeat all analyses reported below by including only reversals devoid of any other indicated reversal in the ± 4 s window. This reduced the number of available epochs considerably leaving only 36.2 ± 24 (mean ± SD) reversals per participant (reflecting 14.8 ± 10.2% of the reported reversals). As this low number of trials does not suffice for reliable statistical results, we opted for a shorter window of ± 2 s around each reversal, which resulted in 131 ± 52.8 trials (38 ± 18%). We repeated all analyses for this reduced dataset and found that all covariations of included parameters and their significance remained the same, with only marginal differences in the numeric values. This suggests that the reported results are not confounded by the presence of an additional indicated in the data epochs.

### Characteristics of perceptual reversal

To characterize the temporal sequence of perceptual reversals, we calculated the interval between subsequent button presses^48,49^. We visually inspected the participant-wise distribution of these intervals and fit them with different distributions (gamma, lognormal, Weibull, exponential, and normal) in MATLAB using the *fitdist* function. Model fit was evaluated using the Akaike Information Criterion (AIC), calculated as 2*k*−2log*L*, where *k* denotes the number of parameters and log*L* the log-likelihood of the model. The distribution with the lowest AIC value across participants was selected to fit the data as shown in Fig. 1B. To test whether one of the two perceptual interpretations of the cube was perceived longer than the other, we calculated the average stability for the left-oriented and right-oriented interpretation and compared these across participants using a paired t-test.

To rule out potential effects of the general time passed during the experiment on the occurrence of reversals, we split the data for each participant into the first and second halves of trials and compared the average number of reversals reported in each half: the difference was not significant (paired t-test; t = −0.406, *p* = 0.690).

### Alignment of respiration and pupil size to perceptual reversals

Previous studies have quantified the alignment between respiration phase and task events using a phase locking value (PLV)^2–5,10,23^. We used this approach to quantify the respiration alignment to perceptual reversals within participants based on the respiration data (sampled at 100 Hz) in epochs of ± 4 s around each reversal. To compute the time course of the within-participant phase locking, we converted the respiration phase at time *t* during epoch *i*, phi(t)_i_, to a complex number. We then computed the average across all epochs within a participant and determined the vector length of this^2,21^:

1 $$\:PLV=\:\left|\frac{1}{N}\sum\:\mathrm{e}\mathrm{x}\mathrm{p}\left(i\:{\phi\:}_{i}\right(t\left)\right)\right|$$

The statistical significance of PLV at the group-level was tested against a null distribution generated from 4000 random temporal shifts of respiration phase under the assumption of no alignment to the experimental task^2,4,5,8^. For each randomization, we derived the maximal value of the group-average phase locking over all time points to correct for multiple comparisons over time. We then calculated the distribution of surrogate PLV values to derive the significance thresholds reflecting *p* < 0.05 and *p* < 0.01.

While we have used this measure of PLV in several previous studies^2,4,5,24^, we note the precise values are statistically biased by the number of included data epochs^50^. This does not affect the comparison of actual and surrogate values in the statistical test, but makes it difficult to compare the phase locking index between different studies and datasets. To allow a better comparison of respiration alignment across studies, we also calculated an alternative measure of phase locking, the so-called pairwise phase consistency (PPC)^50^. This is based on the aggregate angular distances between all pairs of trials.

2 $$\:PPC\left(t\right)=\:\frac{2}{n(n-1)}\sum\:_{i<j}\mathrm{cos}\left({\theta\:}_{i}\left(t\right)-{\theta\:}_{j}\left(t\right)\right)$$

Here *t* is timepoint around the reversal, *n* the number of reversals, and *θi* the respiration phase in radiant for trial *i*. We computed the pairwise phase consistency within participants across all unique reversal pairs (*i < j*) and then averaged this across participants. We calculated the PPC for the current data, and for 12 datasets analyzed in a previous publication from our lab^4^. The 12 datasets described there tested respiration alignment in different visual and auditory paradigms and produced PPC values from 0.015 to 0.087 (0.048 ± 0.023, mean ± SD across datasets). More specifically, the PPC values for the studies described in^4^ are as follows, with the nomenclature taken from that publication: ‘Pitch 1’ (0.056), ‘Time’ (0.057), ‘Visual shape’ (0.087), ‘Emotion 1’ (0.060), ‘Emotion 2’ (0.059), ‘Pitch 2’ (0.044), ‘Arithm’ (0.079), ‘Visual dots’ (0.015), ‘Pitch 3’ (0.021), ‘Pitch 4’ (0.027), ‘Sound’ (0.049), ‘Emotion 3’ (0.020). Unlike the PLV, the PPC is not biased by the number of data epochs included and can be considered a measure of effect size, in a statistical sense, for the coherence of respiratory phase across trials. Because the PPC is computationally more demanding than the PLV, we relied on the PLV for statistical testing.

We also tested for a correlation between the within-participant phase coherence (at the time of maximum coherence in the participant-average) and the number of reported perceptual reversals.

To analyze pupil size, we first computed the epoch-averaged pupil trace for each participant and then derived the group-average of these. We used a cluster-based permutation scheme to test the null hypothesis of whether the group-average differs from zero (i.e., baseline)^51,52^. The first-level inference was derived based on point-wise t-tests of the group-mean to differ from zero and these first-level effects were thresholded at *p* < 0.05. Significant time bins were clustered based on a minimal cluster size of two and using the maximum as cluster statistics. We then contrasted clusters in the actual data with clusters in a surrogate distribution obtained from 20,000 permutations of the first level effects. We report significant clusters at a second-level significance of *p* < 0.01.

### Analysis of the relations between respiration, pupil size and percept stability

We used linear mixed-effect models (LME) to probe the relation between respiration, pupil and the perceptual reversals. In different models we predicted the stability of the percept prior to each indicated reversal (i.e., the duration between the current and previous reversal), the stability of the subsequent percept (i.e., the duration between the current and next reversal) and pupil size based on these variables.

Stability durations (*StabilityPreceding*,* StabilitySubsequent*) were normalized within participants by their respective average to account for the between participant variability, and log transform before entering the model. The phase of respiration was coded as the sine and cosine (*Sine*,* Cosine*) components of the cyclical phase value, as in previous work^2,4,5,24^, respiration frequency (*RespFreq*) was z-scored within participants. Pupil size (*PupilSize*) was also z-scored within participants. The reversal indicated by participants (*PerceptChange*) was included as a categorical variable (dummy coded; level 1 indicating a reversal from right-sided to left-sided interpretation; level 2 from left-sided to right-sided).

The models were then fit across the data from all perceptual reversals across all participants, including participants and reversal number as random intercepts. Since respiratory phase and pupil size are continuous variables, we fit these models at multiple time points (*t*) during the ± 4 s around each reversal (sampled at 200 ms steps). We used a framework of model comparison to establish the predictive power of each factor of interest, similar to our previous studies^2,4,5^. For this, we calculated a full model containing all variables of interest and a reduced model that left out individual factors for each time point. For example, the full model predicting the stability duration of the preceding percept was as follows:

3 $$\begin{aligned}\:StabilityPreceding\hspace{0.17em}&\sim\hspace{0.17em}\mathrm{R}\mathrm{e}\mathrm{s}\mathrm{p}\mathrm{F}\mathrm{r}\mathrm{e}\mathrm{q}\hspace{0.17em}+\hspace{0.17em}\mathrm{S}\mathrm{t}\mathrm{a}\mathrm{b}\mathrm{i}\mathrm{l}\mathrm{i}\mathrm{t}\mathrm{y}\mathrm{S}\mathrm{u}\mathrm{b}\mathrm{s}\mathrm{e}\mathrm{q}\mathrm{u}\mathrm{e}\mathrm{n}\mathrm{t}\hspace{0.17em}+\hspace{0.17em}\mathrm{P}\mathrm{u}\mathrm{p}\mathrm{i}\mathrm{l}\mathrm{S}\mathrm{i}\mathrm{z}\mathrm{e}\:\left(\mathrm{t}\right)\:+\:\mathrm{P}\mathrm{e}\mathrm{r}\mathrm{c}\mathrm{e}\mathrm{p}\mathrm{t}\mathrm{C}\mathrm{h}\mathrm{a}\mathrm{n}\mathrm{g}\mathrm{e}\hspace{0.17em}+\hspace{0.17em}\mathrm{S}\mathrm{i}\mathrm{n}\mathrm{e}\:\left(\mathrm{t}\right)\:\\& \quad+\:\mathrm{C}\mathrm{o}\mathrm{s}\mathrm{i}\mathrm{n}\mathrm{e}\:\left(\mathrm{t}\right)\:+\:\left(1\:\right|\:\mathrm{p}\mathrm{a}\mathrm{r}\mathrm{t}\mathrm{i}\mathrm{c}\mathrm{i}\mathrm{p}\mathrm{a}\mathrm{n}\mathrm{t})\:+\:(1\:|\:\#\mathrm{r}\mathrm{e}\mathrm{v}\mathrm{e}\mathrm{r}\mathrm{s}\mathrm{a}\mathrm{l})\end{aligned}$$

The reduced model, used to test an effect of respiration frequency, was as follows:

4 $$\begin{aligned}\:StabilityPreceding\hspace{0.17em}&\sim\hspace{0.17em}\mathrm{S}\mathrm{t}\mathrm{a}\mathrm{b}\mathrm{i}\mathrm{l}\mathrm{i}\mathrm{t}\mathrm{y}\mathrm{S}\mathrm{u}\mathrm{b}\mathrm{s}\mathrm{e}\mathrm{q}\mathrm{u}\mathrm{e}\mathrm{n}\mathrm{t}\hspace{0.17em}+\hspace{0.17em}\mathrm{P}\mathrm{u}\mathrm{p}\mathrm{i}\mathrm{l}\mathrm{S}\mathrm{i}\mathrm{z}\mathrm{e}\:\left(\mathrm{t}\right)\:+\:\mathrm{P}\mathrm{e}\mathrm{r}\mathrm{c}\mathrm{e}\mathrm{p}\mathrm{t}\mathrm{C}\mathrm{h}\mathrm{a}\mathrm{n}\mathrm{g}\mathrm{e}\hspace{0.17em}+\hspace{0.17em}\mathrm{S}\mathrm{i}\mathrm{n}\mathrm{e}\:\left(\mathrm{t}\right)\:\\& \quad+\:\mathrm{C}\mathrm{o}\mathrm{s}\mathrm{i}\mathrm{n}\mathrm{e}\:\left(\mathrm{t}\right)\:+\:\left(1\:\right|\:\mathrm{p}\mathrm{a}\mathrm{r}\mathrm{t}\mathrm{i}\mathrm{c}\mathrm{i}\mathrm{p}\mathrm{a}\mathrm{n}\mathrm{t})\:+\:(1\:|\:\#\mathrm{r}\mathrm{e}\mathrm{v}\mathrm{e}\mathrm{r}\mathrm{s}\mathrm{a}\mathrm{l})\end{aligned}$$

These models were fit using *fitlme* function in MATLAB (with the default fitting method maximum likelihood). We derived the Akaike Information criterion (AIC) for the full and the reduced models and used their difference for model comparison. From these, we derived the conditional probability of each model given the data, which is known as the Akaike weights^53^. An AIC difference of 9.2 corresponds to a probability of above 99% that the model including the variable of interest has more explanatory power than the reduced model, and an AIC difference of 6 corresponds to a probability of 95%. We report these probabilities as AIC weights. In addition to relying on AIC criteria to determine the relevance of individual predictors, we also report the significance of the slopes of individual predictors. However, we note that for respiration phase, which is a cyclic variable and reflected by both a sine and cosine term, interpreting the significance of each term can be misleading, as strong predictive power is effectively captured by both terms combined. Here, the AIC difference serves as our main criterion to determine the predictive power of respiratory phase, as in previous studies^2,4,5^.

Importantly, these models capture the dependency between each predictor and the dependent variable in the context of all predictors. As a result, also the slopes of the time-independent predictors (e.g. respiration frequency) and their model evidence are also effectively time dependent. For some predictors this time dependency was minimal (e.g. of *StabilityPreceding*, *StabilitySubsequent* or *Percept*) and we report only a single number. For respiration frequency this context dependency was more pronounced and we report the model evidence at all time points (Fig. 3).

## Results

**Fig. 2:**
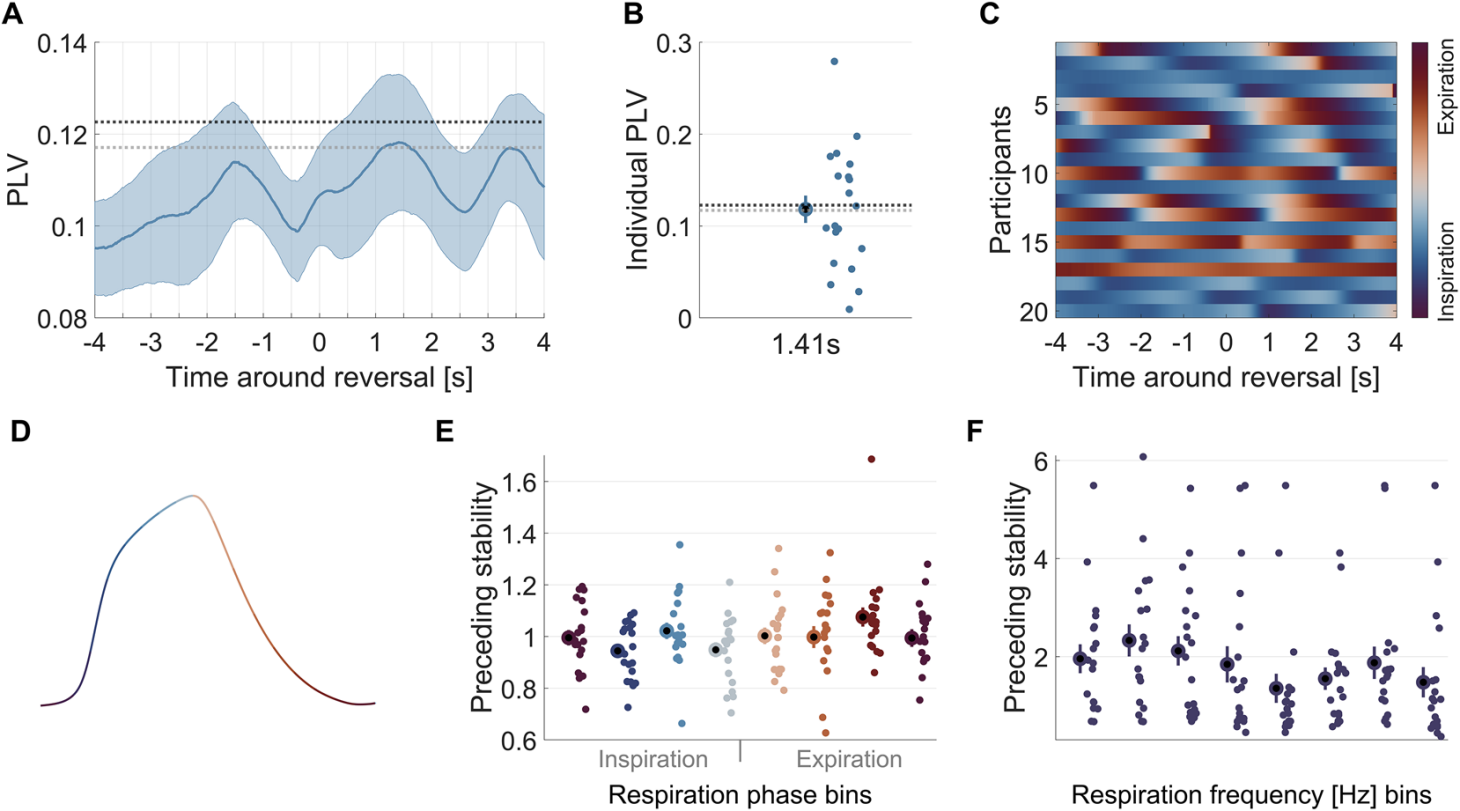
Respiration phase and frequency around perceptual reversals. (**A**) Within-participant phase locking of respiration phase (PLV; mean ± SEM). Dotted lines indicate significance level at *p* < 0.01 (black) and *p* < 0.05 (grey). Phase locking exceeded *p* < 0.05 from 1.15 to 1.61 s. (**B**) Participant-wise PLV at the time point of maximum phase locking in the group average. Dotted lines as in panel A. (**C**) Epoch-averaged respiration phase for each participant. (**D**) The schematic of a respiratory trace indicates the color-coding of the respiratory cycle. (**E**) Normalized duration of the percept preceding each indicated reversal as a function of binned respiration phase. Bold dots show the group average with errorbars showing the SEM, smaller dots represent individual participants. (**F**) Normalized duration of the preceding percept as a function of the (normalized) respiration frequency. *N* = 20.

The average perceptual stability for the 20 participants included in the analysis was 3.96 ± 2.93 s (mean ± SD). The left-oriented interpretation was on average perceived for a longer period (4.73 ± 1.79 s, mean ± SD) compared to the right-oriented interpretation (3.16 ± 1.23 s, mean ± SD; Fig. 2). For both interpretations, a lognormal distribution fit the across-participant data best (Fig. 1B), and also the participant-wise data (18 participants lognormal, 2 participants gamma). The average duration of respiration cycles was 3.76 ± 0.73 s (mean ± SD), corresponding to a respiration frequency of (0.28 ± 0.05 Hz). Inspiration phases were slightly shorter (1.71 ± 0.43 s) than expiration phases (2.09 ± 0.43 s).

### Respiration is only weakly aligned to perceptual reversals

To test whether participants’ respiration phase was systematically aligned to perceptual reversals, we computed the respiration phase-locking value (PLV) for each participant. This within-participant phase-locking was significant at time points subsequent to the indicated reversal (at *p* < 0.05; corrected for multiple comparisons along time; 1.15 to 1.61 s; Fig. 2A, grey dotted line). However, this effect did not reach a more stringent criterion of *p* < 0.01.

To be able to compare the strength of this respiration alignment locking to previously published data, we also computed a second measure of phase locking, which is more robust to differences in the number of data epochs included in the analysis, and hence can be considered a more robust estimate of an actual effect size (the pairwise phase coherence; PPC). For the present data this effect size of respiratory phase locking was much smaller (PPC: 0.011 ± 0.002, mean ± SEM across participants) compared to values obtained for multiple other data sets testing the alignment to external sensory events^4,5,24^: there, across 12 datasets, the alignment to the trial-wise response ranged between 0.015 and 0.087, with an average of 0.048 ± 0.023 (mean ± SD). Hence in the current data the respiratory phase locking is both considerably weaker compared to previous reports and at the edge of significance. In line with a group-level effect that is not very robust, the individual-participant data shown in Fig. 2B reveal considerable heterogeneity, which is also reflected in the participant-wise epoch-averaged respiratory phase (Fig. 2C). We also computed the correlation of the total number of reported reversals and the within-participant phase coherence across participants. This was not significant (*r* = −0.392, *p* = 0.087).

### Respiration frequency but not phase explains percept stability

To understand whether respiration, pupil and the individual percepts are predictive of each other, we used a framework of linear modelling. Using this, we first predicted the percept stability based on the combined respiratory and pupil data and the individual reported interpretation of the cube (i.e. the specific button participants pressed). We base our interpretation of the model outcome on both the significance of the respective predictors and model comparison based on AIC criteria (see Methods). The results for the full models are reported in Table 1.

**Fig. 3:**
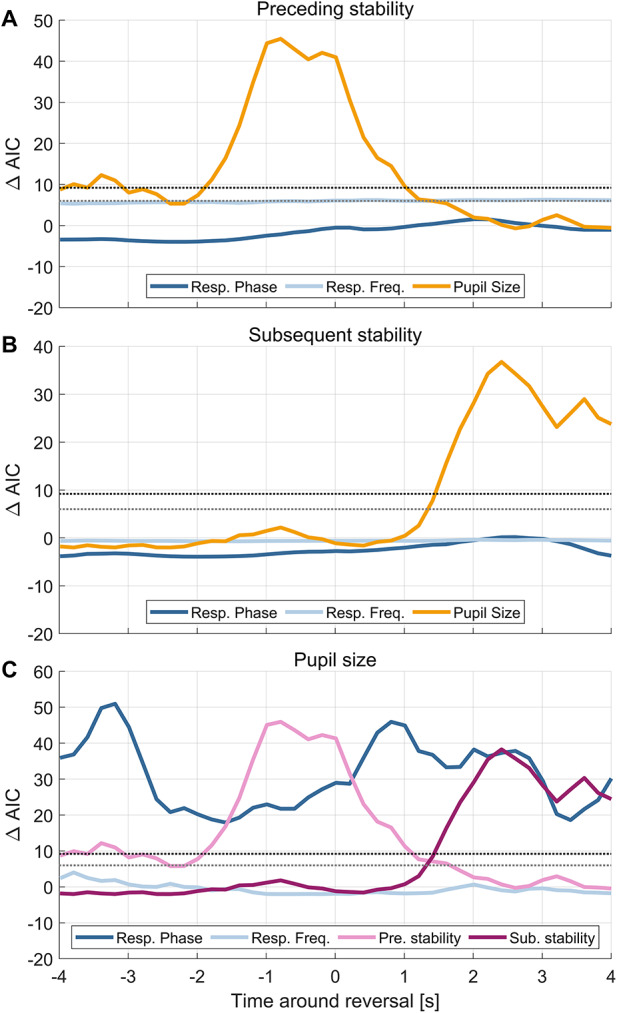
Model comparison results. The graphs show the group-level AIC differences of the full and reduced models testing the influence of individual factors. Positive values correspond to a higher explanatory power of the full model; dotted lines indicate the Akaike weights reflecting a chance of 95% (grey) and 99% (black) that the full model outperforms the reduced models. Time points of maximal differences are reported in Table 1. (**A**) Predictive power of respiration phase, respiration frequency, and pupil size on the stability of the preceding percept. (**B**) Predictive power of respiration phase, respiration frequency, and pupil size on the stability of the subsequent percept. (**C**) Predictive power of respiration phase, respiration frequency, preceding stability, and subsequent stability on pupil size.

Based on the model comparison we found that respiratory phase does not have predictive power for either the stability of the preceding or subsequent precept, at any time point in the epoch around each reversal (Fig. 3A; preceding percept: max deltaAIC = 1.54 at 2.0 s, AIC weight = 0.684; Fig. 3B; subsequent percept: max deltaAIC = 0.16 at 2.4 s, AIC weight = 0.521). To illustrate this data, Fig. 2E displays the participant-wise (within-participant normalized) duration of the preceding percept against the binned respiration phase at the time of the indicated reversal.

**Table 1:** Results of the full linear mixed effect models. The table reports fixed-effect estimates (β), their standard errors (SE), t-values (calculated as the ratio of the estimate to its standard error), and corresponding p-values indicating statistical significance (α = 0.05). Rows denote the dependent variables, and columns denote the predictors. See Eq. 3 in the Methods.

	Respiration phase	Respiration frequency	Preceding stability	Subsequent stability	Pupil size	Percept
Preceding stability	*Sine*: β = 0.020, SE = 0.011, t = 1.86, *p* = 0.0632 *Cos*: β=−0.016, SE = 0.011, t=−1.48, *p* = 0.138	β=−0.022, SE = 0.008, t=−2.89, *p* = 0.004		β = 0.090, SE = 0.012, t = 7.23, *p* < 10^− 5^	β=−0.053, SE = 0.008, t=−6.92, *p* < 10^− 5^	β=−0.391, SE = 0.016, t=−24.26, *p* < 10^− 5^
Subsequent stability	*Sine*: β=−0.003, SE = 0.011, t=−0.32, *p* = 0.747 *Cos*: β=−0.022, SE = 0.011, t=−2.02, *p* = 0.044	β=−0.010, SE = 0.008, t=−1.28, *p* = 0.202	β = 0.092, SE = 0.013, t = 7.27, *p* < 10^− 5^		β=−0.048, SE = 0.008, t=−6.24, *p* < 10^− 5^	β = 0.380, SE = 0.016, t = 23.47, *p* < 10^− 5^
Pupil size	*Sine*: β=−0.100, SE = 0.018, t=−5.65, *p* < 10^− 5^ *Cos*: β=−0.085, SE = 0.018, t=−4.81, *p* < 10^− 5^	β = 0.031, SE = 0.013, t = 2.45, *p* = 0.014	β=−0.142, SE = 0.020, t=−6.94, *p* < 10^− 5^	β=−0.129, SE = 0.020, t=−6.36, *p* < 10^− 5^		β=−0.090, SE = 0.028, t=−3.20, *p* = 0.001

We also tested whether the respiration frequency is predictive of the percept durations. For the duration of the preceding percept this was significant (max deltaAIC = 6.30, AIC weight = 0.959, at 3.0 s) and the slope of this predictor was significant and negative, indicating that higher respiration frequency is associated with a shorter percept stability (Table 1). Figure 3A illustrates the individual data for this effect. Interestingly, for the duration of the subsequent percept, respiration frequency did not have significant predictive power (max deltaAIC = −0.37, AIC weight = 0.453 at 2 s; Fig. 3B) and the slope of this predictor was not significant (Table 1).

These models also corroborate a significant difference in the stability of the two interpretations of the cube, as the respective perceptual interpretation was a significant predictor for both the preceding percept (max deltaAIC = 531.43, AIC weight = 1; Table 1) and the subsequent percept (max deltaAIC = 498.26, AIC weight = 1; Table 1). Furthermore, the stability of the two percepts was related to each other (max deltaAIC = 49.60, AIC weight = 1; Table 1) suggesting that generally faster (or slower) reversals in perception tended to follow each other.

### Pupil size changes around reversals and predicts percept stability

**Fig. 4:**
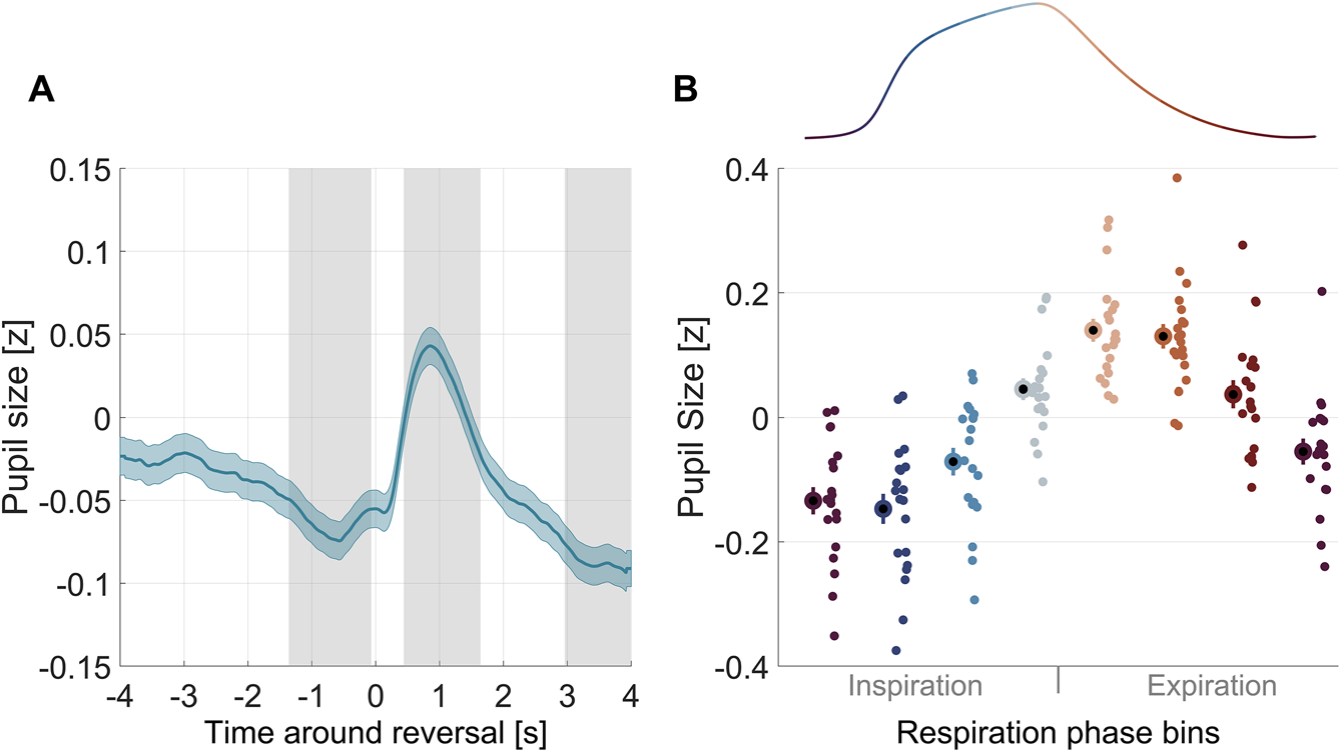
Pupil size around perceptual reversals. (**A**) Group-averaged pupil trace around perceptual reversals (mean ± SEM). Grey shaded areas show epochs in which the pupil size differs significantly from zero (cluster-based permutation test, one cluster from − 1.36 to −0.07 s, *p* = 0.019; one cluster from 0.44 to 1.64 s, *p* = 0.019; one cluster from 2.96 to 4 s, *p* = 0.039). (**B**) Pupil size as a function of respiration phase (8 bins) in the epoch around reversal. Bold dots show the group average with errorbars showing the SEM, smaller dots represent individual participants. Respiration phase has predictive power on pupil size (full model: *p* < 10⁻⁵) as reported in Table 1.

In line with previous studies^32,33,37^, we found that the pupil systematically constricts preceding the reversal and dilates shortly after the reversal (Fig. 4A). This modulation of pupil size was statistically significant (cluster-based permutation test, one cluster from − 1.36 to −0.07 s, *p* = 0.019; one cluster from 0.44 to 1.64 s, *p* = 0.019; one cluster from 2.96 to 4 s, *p* = 0.039, Fig. 4A grey areas). Similar as for respiration, we then asked whether pupil size is predictive of the stability of the individual percepts. This was the case for the preceding percept at time points prior to the indicated reversal (max deltaAIC = 45.47, AIC weight = 1 at −0.8 s, Fig. 3A; Table 1) and for the subsequent percept at time points after the indicated reversal (max deltaAIC = 36.75, AIC weight = 0.999 at 2.4 s, Fig. 3B; Table 1).

### Pupil size changes with respiration phase

Finally, we asked whether respiration is predictive of pupil size, as suggested by previous work^13,38^. This revealed significant predictive power of respiration phase (Fig; 3 C; max delta AIC = 50.44, AIC weight = 1 at −3.2 s). Figure 4B shows the relationship between pupil size along the respiration cycle, with the pupil being smallest around inspiration onset and largest around expiration onset.

Respiration frequency did not offer significant predictive power on pupil size (max deltaAIC = 4.01, AIC weight = 0.881 at −3.8 s, Fig. 3C), although the slope of respiration frequency was statistically significant (beta = 0.031, *p* = 0.014; Table 1). The indicated reversal had significant predictive power on pupil size (max deltaAIC = 8.20, AIC weight = 0.984 at 0.8 s) with perceptual reversal from the right-sided to the left-sided interpretation of the cube being associated with a larger modulation of the pupil (beta = −0.090, *p* = 0.001; Table 1). Finally, and in line with the above results, the stability of each percept was predictive of pupil size (preceding percept: max deltaAIC = 45.94, AIC weight = 1; subsequent percept: max deltaAIC = 38.27, AIC weight = 1, Fig. 3C).

All in all, these results confirm the previously reported variation of pupil size with respiration phase, but also show that in the present paradigm pupil size is also related to the nature and stability of the bistable percept.

## Discussion

The present study examined the three-fold relation between respiration, pupil size and spontaneous changes in perception. Our data reproduce the previously reported modulation of pupil size around perceptual reversals and the general covariation of pupil size with respiration phase. However, our data suggest only a weak, if any, alignment of respiration phase to perceptual reversals. The respiration phase was also not predictive of the stability of the perceptual periods, although respiration frequency was. Overall, this suggests that there is no systematic and strong relation of respiration phase with spontaneous changes in perception, unlike in externally driven sensory-cognitive tasks where respiration predictively aligns with upcoming experimental trials.

### Respiration phase is only weakly aligned to perceptual reversals

While previous studies using externally-driven sensory-cognitive tasks report a robust alignment of respiration phase to individual trials^1–5,9,18–24^, endogenously produced changes in perception seem to lack this alignment. For the present paradigm, the phase locking strength was weak when compared to that reported in previous studies testing paradigms based on external stimuli^3–5,24^, and reached only about a fourth of the magnitude found in previous studies (based on the PPC). Although statistical significance is not a measure of effect size, the residual observed effect reached only a moderate level of significance (*p* < 0.05), while previous studies typically found highly significant respiration alignment.

Although studies using stimulus-driven paradigms report strong respiration alignment, the factors driving this remain unclear. The reduced phase alignment seen here suggests that the temporal expectation of an external signal, or the expected needed to act on this, chiefly shapes respiration alignment. Indeed, in the externally-driven tasks, alignment was found to emerge already 2–3 s prior to individual trials, suggesting that respiration actively aligns to the upcoming and expected events^4,24^. In support of this, recent work suggests that precisely this external temporally predictive information is critical to facilitate alignment, as both spatial cues and temporal cues were shown to facilitate alignment and increase perceptual sensitivity^25^. In line with this, the alignment to endogenously driven changes in perception is then best explained by a lack of an externally paced expectation of when perception will change.

Still, we did observe weak respiration alignment. The strongest effect was observed following the indicated perceptual reversal, pointing to the motor act of reporting the change in percept as key driver of this, potentially facilitating the transient synchronization of respiration to the motor act^3^. In support of this, it seems that the need for a motor action is a critical factor shaping the large-scale engagement of many brain regions during spontaneous changes in perception^54^. Perhaps then, in paradigms devoid of motor actions the respiration alignment would be even more reduced. Clearly, future studies using other (e.g. no-report) paradigms are required to directly test how far motor actions contribute to respiration alignment. All in all, our results support that temporal prediction of external events is a key driver of respiration alignment, but also show that ultimately alignment is driven by multiple factors.

We note that the reduced alignment in the present data cannot be explained by a different time scale of the perceptual events during bistable perception and the externally-driven sensory tasks in previous work^4,5,24^. The average stability of individual percepts reported here was comparable to the average duration of respiratory cycles of the participants (both between 3.5 and 4 s). In the externally-driven tasks analyzed previously, the time scale of the typical stimulus-to-stimulus intervals was also comparable to the typical duration of the participants there^4^(both around 3.5 s; Fig. 1 there), and was comparable to the stability of individual percepts here. Hence, the intrinsic time scales of respiration and perceptual events are comparable in both types of paradigms and cannot explain the strong differences in respiration alignment.

### Respiration frequency relates to perceptual reversals

While the respiration phase is only weakly aligned to perceptual reversals, it is possible that perception in general is still entrained by the rhythm of respiration. In support of this, we observed that respiration frequency is predictive of the duration of the perceptual stability, in particular of the percept that is present during the respiratory cycle used to define the momentary respiration frequency: there, higher respiration frequency was associated with a shorter stability of the percept. And as noted above, both changes in perception and respiratory cycles are of the same time scale. This suggests that the overall pace of respiration and that of perceptual reversals may be related. While the present data cannot disentangle their causal relation, whether respiration frequency paces reversals in perception, or vice versa, the general relation points to a link between cycles in perception and cycles in respiration. However, the typical variability of the duration of bistable percepts tends to follow a skewed and wide distribution^27,33,43^, while the variability in respiration rate is much smaller, hence any such relation is not straightforward. Future studies employing manipulations of participants’ respiration frequency, or of manipulations of the stability of bistable percepts^55–57^, could be used to investigate this relation between respiration frequency and perceptual cycles further.

### The slow dynamics of perceptual reversals may limit respiration alignment

It could also be that the alignment of respiration to perceptual reversals is shadowed by the temporal variability and sluggishness of the underlying neural processes. Neuroimaging studies support that paradigms involving bistable perception engage higher-order brain networks comprising frontoparietal and temporal areas^26,27^, in particular when motor responses are involved^54^. These networks reflect the integration of feedforward and feedback signals^28^ and meta-analytic studies support the view that reversals arise from gradual, distributed interactions across multiple hierarchical levels^29^. Neural activity in those regions evolves over slower timescales than the activity in early sensory regions^40–43^. Hence, perceptual reversals are accompanied by widespread and relatively slow and gradual transitions of brain activity, rather than brief and immediate changes that are often associated with sudden external signals^40^. On the other hand, many studies have shown that respiration is related to the large-scale dynamics of neural activity^6–8,58^. This alignment of task-relevant neural processes to respiration^1,5,9–12^ may also explain the observed alignment of respiration to sensory tasks, at least when the relevant neural processes are tightly aligned to the external events. However, the slow buildup and variability of neural transitions underlying perceptual reversals^41,42^ may lack the sharp and reliable onset necessary to result in a precise alignment of respiration to perceptual events, simply via the alignment of the relevant neural processes to these. Future studies directly including measurements of neural activity during bistable perception are required to further understand this.

### Pupil size changes systematically around reversals

We observed a consistent modulation of pupil size locked to perceptual reversals, with a constriction preceding the reported changes in perception and a pronounced dilation following this. This bi-phasic change in pupil size corroborates previous studies. Following these, the pupil constriction prior to a reversal may reflect the action of the noradrenergic arousal system on neural representations of the different interpretations of the bistable image^30–33^. In favor of arousal shaping pupil dynamics, our data support more pronounced changes in pupil for perceptual reversals from the right-oriented to the left-oriented cube. The left-oriented cube corresponds to what is also known as the ‘from-above’ view in the literature, and which tends to be more dominant and perceived as longer compared to the alternative (right-oriented) interpretation^59^. Hence, one could speculate that perception changing towards the dominant interpretation of the Necker cube is accompanied with larger changes in arousal.

However, the changes in pupil size may in part also reflect task requirements and motor-related processes, in particular in the epoch after the indicated reversal^30,31,33^. Indeed, previous work has suggested that motor actions may be a key driver of the observed pupil dilation following the reversal^32^. Dissociating both, arousal and motor action, again requires other paradigms, either with more calibrated perceptual interpretations of a bistable figure, direct manipulations of their stability, or paradigms devoid of motor actions^32,59^.

Pupil size was also predictive of the stability of individual percepts. In our data pupil size preceding the reversal was predictive of the stability of the preceding percept, and pupil size after the reversal was predictive of the stability of the following percept. Importantly, this relation persisted when we repeated the analysis by excluding epochs that contained an additional reported reversal in a 2 s epoch, ruling out confounds from additional motor-related processes^31,60,61^. Previous studies have also reported a significant relation between pupil and perceptual stability, although the directionality of the effect remains inconclusive across these^32,37^. Possibly, differences in attentional engagement or arousal between tasks modulate the specific relation, i.e. whether particularly large or small pupil size is predictive of a more stable percept.

### Pupil size is linked to respiration phase

Finally, our data reproduce the covariation of pupil size with respiration phase. Such a covariation of pupil size and respiration phase has been reported during resting periods and cognitively demanding tasks in previous studies^13,14,38^. This may reflect the differential engagement of arousal along the respiration phase, presumably enabled by the bidirectional projections between the locus coeruleus and the preBötzinger complex^62^. One possibility is that temporal variations in arousal, arising from changes in arousal due to motor actions or the nature of the momentary percept, are relayed to the preBötzinger complex and directly shape respiration according relative to the current task. Given that pupil size systematically changes around perceptual reversals, such a driving relation between pupil and respiration could also be responsible for the residual alignment of respiration phase to the reported changes in perception. While our data corroborate the prominent link of pupil size and respiration phase the consequences of this coupling and the combined influence of pupil size and respiration phase on sensation and cognition need to be studied in more detail.

## Data Availability

Data and Matlab code are available here: https://doi.org/10.5281/zenodo.17868201.
